# Southern rice black-streaked dwarf virus: a white-backed planthopper-transmitted fijivirus threatening rice production in Asia

**DOI:** 10.3389/fmicb.2013.00270

**Published:** 2013-09-09

**Authors:** Guohui Zhou, Donglin Xu, Dagao Xu, Maoxin Zhang

**Affiliations:** College of Natural Resources and Environment, South China Agricultural UniversityGuangzhou, China

**Keywords:** Southern rice black-streaked dwarf virus, fijivirus, *Sogatella furcifera*, virus transmission, infection cycle

## Abstract

Southern rice black-streaked dwarf virus (SRBSDV), a non-enveloped icosahedral virus with a genome of 10 double-stranded RNA segments, is a novel species in the genus *Fijivirus* (family *Reoviridae*) first recognized in 2008. Rice plants infected with this virus exhibit symptoms similar to those caused by *Rice black-streaked dwarf virus*. Since 2009, the virus has rapidly spread and caused serious rice losses in East and Southeast Asia. Significant progress has been made in recent years in understanding this disease, especially about the functions of the viral genes, rice–virus–insect interactions, and epidemiology and control measures. The virus can be efficiently transmitted by the white-backed planthopper (WBPH, *Sogatella furcifera*) in a persistent circulative propagative manner but cannot be transmitted by the brown planthopper (*Nilaparvata lugens*) and small brown planthopper (*Laodelphax striatellus*). Rice, maize, Chinese sorghum (*Coix lacryma-jobi*) and other grass weeds can be infected via WBPH. However, only rice plays a major role in the virus infection cycle because of the vector’s preference. In Southeast Asia, WBPH is a long-distance migratory rice pest. The disease cycle can be described as follows: SRBSDV and its WBPH vector overwinter in warm tropical or sub-tropical areas; viruliferous WBPH adults carry the virus from south to north via long-distance migration in early spring, transmit the virus to rice seedlings in the newly colonized areas, and lay eggs on the infected seedlings; the next generation of WBPHs propagate on infected seedlings, become viruliferous, disperse, and cause new disease outbreaks. Several molecular and serological methods have been developed to detect SRBSDV in plant tissues and individual insects. Control measures based on protection from WBPH, including seedbed coverage, chemical seed treatments, and chemical spraying of seedlings, have proven effective in China.

## INTRODUCTION

Southern rice black-streaked dwarf disease (SRBSDD) was first discovered in Yangxi County, Guangdong Province, China, in 2001, and its occurrence was limited to southern China from 2002 to 2008 (Zhou et al., 2004, 2008; Wang et al., 2010a). However, by 2009, the disease had spread to 19 provinces in northern Vietnam and nine provinces in southern China, where it damaged 42,000 ha and more than 300,000 ha of rice (*Oryza sativa*) fields, respectively. In 2010, more than 60,000 ha of paddy fields in 29 provinces of Vietnam and more than 1,300,000 ha in 13 provinces of China became infected, causing crop failure in many places (Zhou et al., 2010; Hoang et al., 2011; Zhai et al., 2011). Although great efforts were undertaken to control the disease, nonetheless, it damaged more than 700,000 ha in 2011 and over 500,000 ha in 2012 in the two countries. Moreover, the disease has now been found in some areas in Japan (Matsukura et al., 2013).

## SYMPTOMS AND CHARACTERISTICS OF SRBSDD

**FIGURE 1:**
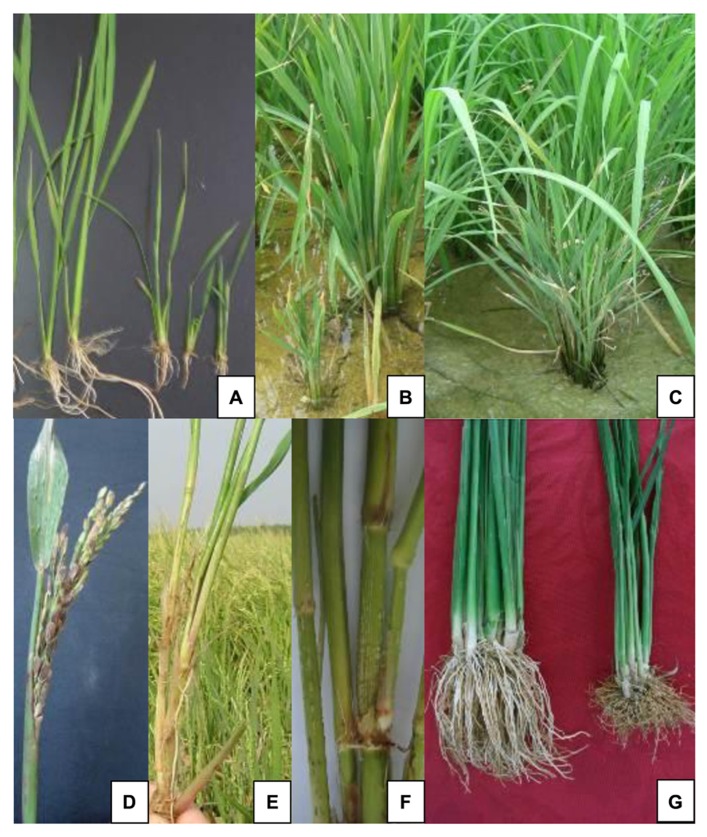
**Symptoms of southern rice black-streaked dwarf disease.** **(A)** Very early infected seedlings (right) showing dwarfism and stiff leaves. **(B)** Diseased plant (front) infected at the seedling stage showing severe stunting and withering after transplanting. **(C)** Diseased plant (front) infected at the early tillering stage showing dwarfism and excessive tillering. **(D)** Diseased head showing small spikes and barren grains. **(E)** Aerial rootlets and branches on the stem nodes of an infected plant. **(F)** Small streaked white or black waxy galls on the stems of an infected plant. **(G)** Poorly developed brown roots (right) of an infected plant.

Rice is susceptible to SRBSDD during all growth periods, but the symptoms and yield losses depend on the growth stage at the time of infection. The earlier the infection occurs, the more severe the symptoms (Zhou et al., 2010). (1) Plants infected at the early seedling stage exhibit dwarfism and stiff leaves (**Figure 1A**). Most symptoms are not obvious in the seedbed; they usually appear after the rice plants have been transplanted to the paddy field. Severe stunting (shorter than one third of the normal height) and failure to elongate occur, and seriously-diseased plants may die of withering (**Figure 1B**). (2) Infection at the early tillering stage results in significant dwarfing, with excessive tillering and failure to head (**Figure 1C**). (3) Infection at the elongating stage does not stunt the plants, but they produce small spikes, barren grains, and deficient grain weight (**Figure 1D**). The diseased leaves are short, dark green, and rigid, and ruffles often appeared near the leaf base on the surfaces of upper leaves. (4) Plants infected at the booting stage show no visible symptoms, but the infection can be confirmed by enzyme-linked immunosorbent assay (ELISA) or reverse-transcription polymerase chain reaction (RT–PCR). After elongation, the plants infected before the tillering stage usually display the most common symptoms of this disease, including dark green foliage; aerial rootlets and branches on the stem nodes (**Figure 1E**); small, streaked, black or white, waxy galls 1–2 mm in size on the stems (**Figure 1F**); and poorly-developed brown roots (**Figure 1G**).

Southern rice black-streaked dwarf disease has the following characteristics (Zhou et al., 2010): (1) it is extensively distributed in rice growing areas, but serious damage occurs only in limited regions in some years. Because the disease spreads via transmission by the white-backed planthopper (WBPH, *Sogatella furcifera*), a long-distance migratory pest, its distribution coincides with the migration and range of WBPH. However, the disease is severe only when large-scale invasion and propagation of viruliferous WBPHs take place during the seedling stage. The disease severity in a given area is thus closely related to the original sources of the virus and WBPH population, and the sowing time and growth stage of the rice, the cultivation pattern, climate conditions, and the geographic landform.

(2) Southern rice black-streaked dwarf disease affects summer rice more seriously than winter or spring rice. Its severity depends on the timing and extent of immigration of the viruliferous WBPH population. Dwarfism is observed mainly on plants infected early. Therefore, the earlier, extensive WBPH immigration causes more serious effects. Although severely-diseased winter or spring rice plants may be seen occasionally in northern Vietnam and southern Hainan Province, China, the major overwintering places for WBPH, spring rice in most rice growing areas of China is very mildly affected, with less than 1% dwarfed plants in most fields and rarely with 3–5% in several fields. However, infected spring rice plants may serve as an infection source for summer rice growing in the same or other places. In most rice growing areas in China, the infection rates steadily increase through the growing seasons, i.e., from low on spring rice to high on early and late summer rice. In southern Japan, SRBSDD is observed yearly after mid-June to mid-July, when the overseas immigration of WBPH from China or Vietnam occurs (Matsukura et al., 2013).

(3) Southern rice black-streaked dwarf disease afflicts hybrid rice more than inbred rice, as indicated by field surveys. This difference is probably due to both the higher compatibility between hybrid rice and WBPH, which may increase infection opportunities, and the single seedling transplanting of hybrid rice, which eliminates compensation from healthy plants.

(4) Normally, in fields with low SRBSDD occurrence rates (below 3%), diseased plants are dispersed and have mild symptoms, while in fields with high rates (above 10%), infected plants are clumped and have severe symptoms. These clumps are always surrounded by many plants with milder symptoms (darkened leaves and galls on stalks, but no stunting). A similar distribution pattern also occurs when a “severe disease” field is neighbored by “mild disease” fields and may result from the relatively slow dispersal (and virus transmission) of the viruliferous WBPH nymphs that develop on early-infected rice plants.

## PATHOGEN AND TRANSMISSION

**FIGURE 2:**
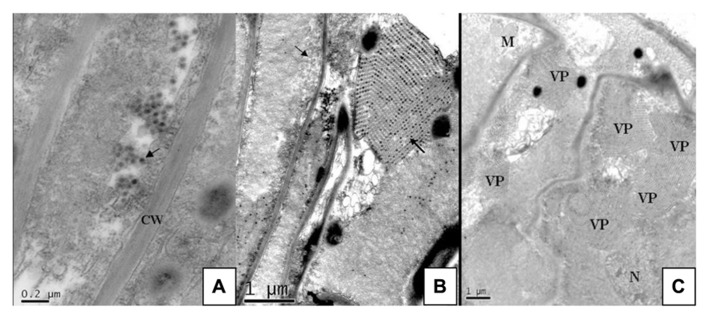
**Ultra-thin sections of rice vein cells infected with SRBSDV. From Zhou et al. (2008).** **(A)** Separate virus particles in the cytoplasm near the cell wall (arrow). **(B)** Virus particles arrayed in rows inside a transverse tubule (arrow) and numerous virus particles aggregated into crystalline array in the viroplasms (arrow). **(C)** Viroplasms full of virus scattered in the cytoplasm. CW, cell wall; N, nucleus; VP, viroplasm; M, mitochondrion.

**FIGURE 3:**
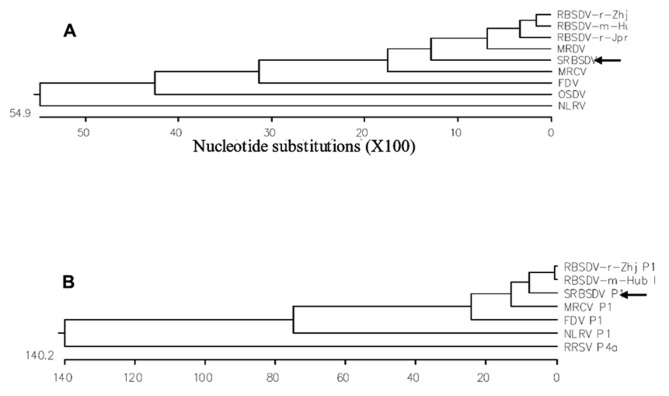
**Polygenetic trees based on S10 nucleotide sequences (A) and P1 amino acid sequences (B) of SRBSDV and other related fijiviruses.** From Zhou et al. (2008) andWang et al. (2010a). To construct the phylogenetic trees, viral nucleotide and amino acid sequences were obtained from GenBank, aligned using MegAlign, and analyzed using the neighbor-joining method (). S10 nucleotide sequence accession numbers are EU523360, NC_003733, D00606, AF227205, L76560, AY607586, NC_007162, AB011025, and NC-003653 for SRBSDV, RBSDV-r-Zhj, RBSDV-r-Jpn, RBSDV-m-Hub, MRDV, MRCV, FDV, OSDV, and NLRV, respectively. P1 amino acid sequence accession numbers are FN563983, NC_003729, AY144568, AF499925, NC_007159, NC_003654, and NC_003771 for SRBSDV, RBSDV-r-Zhj, RBSDV-m-Hub, MRCV, FDV, NLRV, and RRSV, respectively.

The viral pathogen of SRBSDD is currently a tentative species in the genus *Fijivirus*, family *Reoviridae *(Zhou et al., 2008; Wang et al., 2010a). The name Southern rice black-streaked dwarf virus (SRBSDV) was proposed because it resembles the *Rice black-streaked dwarf virus* (RBSDV) in disease symptoms, viral particles, and genome structure; the word “Southern” refers to southern China (where it was first identified) and the southern part of the Northern Hemisphere where it is currently distributed (RBSDV occurs in the northern part of that hemisphere). The virions of SRBSDV are icosahedral particles 70–75 nm in diameter that appear only in the phloem of infected plants, while the virus aggregates into a lattice structure in the cells (**Figure 2**). The viral genome contains ten linear double-stranded RNA (dsRNA) segments that range in size from approximately 4.5 to 1.8 kb and are named S1–S10 according to their molecular weights (from large to small; Zhou et al., 2008; Wang et al., 2010a). Sequence data indicate that SRBSDV is most closely related to *maize rough dwarf virus* (MRDV), followed by RBSDV and *Mal de Río Cuarto virus* (MRCV; **Table 1**; **Figure 3**).

**Table 1:** Identities (%) of nucleotide and predicted amino acid (bracketed) sequences between SRBSDV Hainan isolate and other plant-infecting fijiviruses^*^.

Segment	RBSDV-r-Zhj	RBSDV-m-Hub	MRCV^**^	MRDV	FDV	OSDV
S1	79.0 (85.8)	79.0 (86.1)	74.1 (78.6)	n/a^***^	67.3 (63.7)	n/a
S2	78.9 (89.0)	79.2 (89.0)	75.7 (83.1)	n/a	62.2 (59.4)	n/a
S3	73.0 (73.4)	73.1 (73.8)	65.5 (56.8)	n/a	53.8 (37.5)	n/a
S4	77.3 (84.5)	77.3 (84.5)	71.1 (72.9)	n/a	63.2 (53.6)	n/a
S5	70.6 (69.9, 61.8)	71.6 (67.7, 62.7)	66.3 (59.1, 50.5)	n/a	51.1 (30.9, –)	n/a
S6	70.6 (63.1)	70.7 (63.3)	62.4 (42.5)	n/a	11.2 (23.2)	n/a
S7	73.3 (80.7, 60.6)	73.2 (80.7, 60.6)	65.5 (61.7, 42.3)	74.2 (82.7, 61.5)	59.7 (52.5, 25.2)	22.8 (34.1, 10.2)
S8	72.6 (71.3)	72.5 (70.6)	64.2 (55.4)	72.6 (70.3)	40.3 (35.8)	15.1 (22.6)
S9	74.2 (77.4, 72.0)	74.0 (77.1, 72.0)	68.8 (67.1, 61.6)	74.9 (77.4, 73.5)	51.3 (37.1, 37.8)	40.5 (26.3, 18.0)
S10	78.5 (83.4)	78.8 (84.3)	72.5 (71.9)	79.1 (83.5)	56.4 (47.6)	45.4 (33.1)

* From Wang et al. (2010a).

** MRCV, Mal de Río Cuarto virus; MRDV, maize rough dwarf virus; FDV, Fiji disease virus; OSDV, oat sterile dwarf virus.

*** n/a, no sequence available

The SRBSDV genome contains 13 open reading frames (ORFs): S5, S7, and S9 each encode two proteins (P5-1, P5-2, P7-1, etc.), while the other seven segments each encode one. Genomic comparisons have shown that S1–S4, S8, and S10 of SRBSDV encode putative structural proteins: the RNA-dependent RNA polymerase, major core structural protein, capping enzyme, outer shell B-spike protein, minor core protein, and major outer capsid protein, respectively; P6, P7-1, P7-2, P9-1, and P9-2 encode putative non-structural proteins (Zhang et al., 2008; Zhou et al., 2008; Wang et al., 2010a). Several studies have investigated the functions of these proteins. The P6 is an RNA silencing suppressor (Lu et al., 2011) and might participate in viroplasm formation by interacting with itself and P5-1 (Li et al., 2013a) and by recruiting P9-1 (Wang et al., 2011). However, Mao et al. (2013) found that the P5-1 and P9-1 can form filamentous and granular viroplasm matrices respectively in the WBPH cells without the viral infection, and suggested that the P6 was recruited by direct interaction with these two proteins. Based on their findings, a new model for the genesis and maturation of the viroplasms induced by fijiviruses in insect vector cells was proposed (Mao et al., 2013). The P6 also interacts with a rice translation elongation factor 1A (eEF-1A) to inhibit host protein synthesis (Zhang et al., 2013). The P7-1 has been reported to be the major component of the tubules; it self-interacts to form tubules in insect cells and may be a virus movement protein (Liu et al., 2011). The P9-1 is the major constituent of the viroplasm and is essential for viral replication in insect vectors (Jia et al., 2012a). The functions of other SRBSDV proteins remain unknown.

The genetic diversity and evolutionary mechanisms of SRBSDV were investigated by Li et al. (2013b), who reported putative recombination events and positive selection. Through June, 2013, hundreds of SRBSDV sequences have been deposited in GenBank, including the complete genomic sequences of seven isolates. The SRBSDV genome is highly conserved, with over 97% nucleotide identities among isolates from different geographic regions, perhaps because the virus experiences large-scale transmission from limited overwintering zones via its vector’s annual migration.

The virus can be transmitted highly efficiently by WBPH in a persistent circulative propagative manner (Cao et al., 2011; Pu et al., 2012). A small proportion (less than 5%) of small brown planthoppers (BPHs; *Laodelphax striatellus*) acquire SRBSDV from infected plants under experimental conditions but cannot transmit it (Jia et al., 2012b; Pu et al., 2012), perhaps because the virus cannot pass from the midgut into the hemocoel or into the salivary glands of this insect (Jia et al., 2012b). The virus is not transmitted by the BPH (*Nilaparvata lugens*), leafhoppers, or rice seeds (Pu et al., 2012; Wang et al., 2010b). It propagates in WBPH and is carried by viruliferous vectors throughout their lives but is not transmitted to eggs. Both WBPH nymphs and adults can transmit the virus to plants, and nymphs acquire the virus more readily than adults. About 80% of nymphs propagated on infected rice plants may become viruliferous. The minimum virus acquisition and inoculation periods were 5 and 30 min, respectively, for both WBPH nymphs and adults. The circulative transmission periods of the virus in WBPH range from 6 to 14 days, and most viruliferous individuals have one or more intermittent periods that range from 2 to 6 days (Pu et al., 2012). A single newly-hatched, viruliferous WBPH nymph may infect 22–87 (avg. 48.3) rice seedlings with SRBSDV (Cao et al., 2011), and a single adult can transmit the virus to 8–25 rice plants within 5 days (Pu et al., 2012). WBPH can transmit SRBSDV from rice to maize (*Zea mays*) seedlings, but it rarely acquires the virus from infected maize (Li et al., 2012; Pu et al., 2012).

Southern rice black-streaked dwarf virus could be detected by RT–PCR in all tested rice plant parts except growth point. The virus titer differed significantly among plant parts; the leaf sheath contained the highest SRBSDV titer, followed by the oldest green leaf. The virus titer in the leaf sheath varied significantly with plant age and increased substantially 20 days after infection (Matsukura et al., 2013). The expression levels of most viral genes (except P1) were relatively low on day 14 after infection, rapidly increased from day 14–20, decreased by day 30, increased by day 40, and remained relatively stable by day 50. In contrast, the expression of P1 was significantly down-regulated on days 14 and 20 (He et al., 2013). The virus titers in leaf sheaths and old green leaf correlated positively with WBPH virus acquisition efficiency (Matsukura et al., 2013).

Southern rice black-streaked dwarf virus can naturally infect a number of grasses, including rice, maize, Chinese sorghum (*Coix lacryma-jobi*), *Avena fatua, Echinochloa crusgalli*, *Eleusine indica*, and *Pennisetum flaccidum *(Zhou et al., 2008; Zhu et al., 2012). Diseased maize plants are clearly stunted with swollen internodes; densely clumped and darkened foliage; and wider, shorter, fragile leaves with ruffles. Most infected maize cultivars produce dot-like wax lining small galls along the abaxial leaf veins, and also some produce them on their stalks. Diseased maize plants have short, small ears and always fail to fruit. On SRBSDV-infected Chinese sorghum, *A. fatua, Echinochloa crusgalli*, and *Eleusine indica*, dark green and ruffled leaves also can be observed (Zhou et al., 2008; Zhu et al., 2012).

## DISEASE CYCLE

**FIGURE 4:**
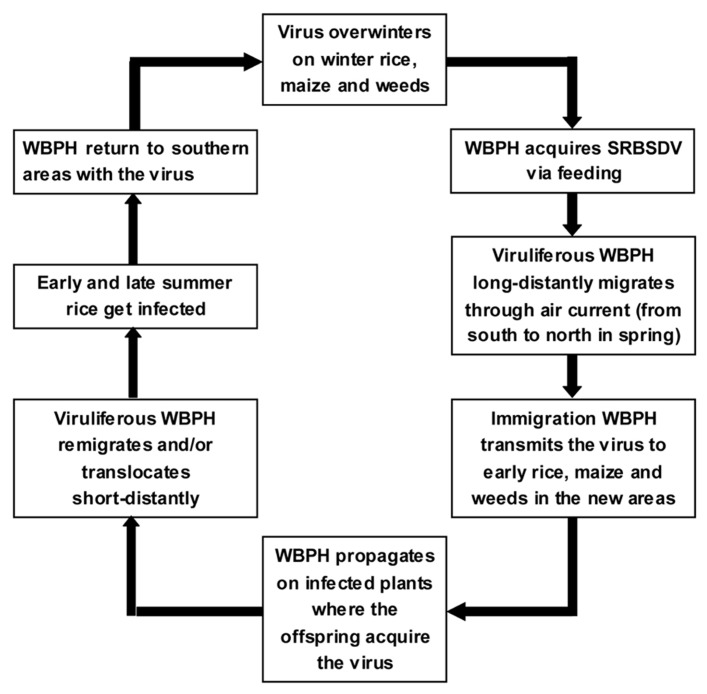
**Infection cycle of Southern rice black-streaked dwarf disease (SRBSDD)**.

In most rice growing areas in China, no winter rice is grown that could host overwintering WBPH. Thus, the primary infection comes from viruliferous WBPH adults migrating northward from their overwintering sites in southern China every spring. The southwestern province of Yunnan and Hainan Island are considered the major WBPH overwintering regions in China. In recent years, the growing area of winter rice on Hainan Island is expanding, providing abundant virus and vector resources. The viruliferous WBPH may migrate into the southern parts of Guangdong and Guangxi provinces and northern Vietnam during February and March. Normally, the viruliferous macropterous adults are driven by the northeastward air flow to the Pearl River Basin (Guangdong) and Honghe Prefecture (Yunnan) in March. Then they migrate to northern Guangdong and Guangxi provinces, Southern Hunan and Jiangxi provinces, and central Guizhou and Fujian provinces in April; to the middle and lower reaches of the Yangtze River and the Huai River Plain during late May to middle or late June; and to the northern provinces and the southern parts of the northeastern provinces, even into Japan, during late June to early July. During autumn, the virus-carrying insects return to their overwintering places in late August when the monsoons shift direction. The SRBSDD cycle (Guo et al., 2010) is illustrated in **Figure 4**.

The occurrence and prevalence of SRBSDD are closely associated with the invasion period and invasion population of WBPH and the percentage of viruliferous individuals. Conditions that favor an epidemic include: a warm winter followed by a rainy early spring; typhoons and strong convection; high mountains, uplands, basins, and river mouths; rice in the seedling and tillering periods; peak immigration of WBPH; and companion planting of single- and double-cropping rice or spring and early/late summer rice.

## SRBSDV–RICE–WBPH INTERACTIONS

Because SRBSDV propagates within its vector, WBPH, it can be considered both a plant virus and an insect virus. Plant viruses have been reported to affect the life parameters of their vectors (Boquel et al., 2012; Czosnek and Ghanim, 2012), and are well documented to influence the vector’s behavior, including host selection, settling and feeding, and dispersal via olfactory cues (i.e., plant volatile organic compounds) that are altered by virus infection (Mauck et al., 2010; Bosque-Perez and Eigenbrode, 2011; Stafford et al., 2011). Tu et al. (2013) revealed that SRBSDV could adversely influence WBPH survival rates, adult longevity, and oviposition rates and that these effects were temperature-dependent. WBPH behavior appears to be altered directly by SRBSDV or indirectly by the infected plants, but more evidence is needed to clarify this relationship. Xu et al. (2012) found that SRBSDV infection may alter WBPH gene expression, involving primary metabolism, ubiquitin-proteasome, cell cytoskeleton organization, and immunity regulatory system pathways. He et al. (2013) revealed by RT–qPCR that all 13 SRBSDV genes showed similar expression patterns in rice, maize, and WBPH, indicating that the virus uses the same infection strategy in plant and insect hosts. Most SRBSDV genes were more abundant in WBPH than in rice and maize, confirming the virus’ higher replication levels in insect hosts than in plant hosts. Across the SRBSDV genome, the mRNA expression levels of P3, P7-1, and P9-2 were highest, and the levels of P6 and P10 were lowest (He et al., 2013).

## PREDICTION AND FORECAST

Similar to other rice viral diseases, SRBSDD occurs occasionally and suddenly with localized devastation and is difficult to forecast. Medium- to long-term predictions must be based on cycles of disease occurrence, disease prevalence, and long-distance WBPH migration, while short-term forecasts should consider cultivation patterns, invasion populations of WBPH, percentages of viruliferous insects, and weather conditions.

(1) Surveys of the base numbers of overwintering diseased plants and pests. Annual surveys from January to March of the infection rates and WBPH population sizes on winter rice in northern Vietnam and Hainan Island are important to predict the occurrence of SRBSDD in most rice producing areas of China, while winter surveys in Northern Burma and southwestern Yunnan province are crucial for predicting outbreaks in the southwest rice production area. Moreover, infection rates of maize growing in WBPH overwintering regions can also provide data for forecasting the disease.

(2) Surveys of the immigration period, invasion population, and percentage of viruliferous WBPH. Severe disease is highly likely when the WBPH immigration period coincides well with the rice seedling stage and where there are large viruliferous invasion populations and many immigration climaxes.

(3) Surveys of the infection rates of spring rice in mid- to late-growth stages and the WBPH population. There is a close correlation between the field infection rate of spring rice and the percentage of viruliferous insects. Viruliferous WBPHs propagated on SRBSDV-infected spring rice may be the infection source for early and late summer rice in the same or other places. Field surveys of the disease and vector populations can provide useful forecasting information.

(4) Surveys of the infection rates of early and late summer rice, the WBPH population, and the percentage of viruliferous individuals. These surveys are especially important for rice production areas in high danger, i.e., areas with past severe occurrences and those in hills, basin, or near river mouths.

(5) Methods for field sampling and virus detection. For survey and detection purposes, whole diseased plants should be collected from fields, their roots cleaned of soil and kept moist with a wet cloth or tissue, and the samples sent to a laboratory for detection. WBPHs can be captured using light traps and stored in a small container with a cotton ball or towel moistened with 70% ethanol to prevent the insects from rotting. To avoid being crushed during shipping, the insects should not be directly soaked in ethanol. Optimally, at least 100 macropterous WBPH adults should be collected for each survey. There are a number of methods for virus detection from plants and insects, such as ELISA (Wang et al., 2012a), dot-immunobinding assay (Chen et al., 2012), Immuno-fluorescence assay (Jia et al., 2012a,b), RT–PCR (Wang et al., 2012b) and reverse transcription-loop-mediated isothermal amplification (RT-LAMP; Zhou et al., 2012). RT–PCR is a proven and highly-reliable method. For example, Wang et al. (2012b) screened 40 pairs of primers and selected two primer sets for a specific and efficient one-step duplex RT–PCR detection method that is commonly used.

## DISEASE CONTROL

Thus far, no SRBSDV-resistant rice cultivar has been found, detailed disease regulation remains unclear, and disease forecast systems and control measures are still under development. Nevertheless, some effective approaches have been devised based on practical experience in disease control in recent years.

(1) Joint prevention and control. Enhanced disease and pest control in their overwintering areas may help prevent the disease from spreading widely. Managing WBPH populations in southern China (where the pest and virus propagate in early spring) may alleviate occurrences of the disease in the northern rice growing areas, while control of the disease and pest on spring rice may reduce the invading pest populations and virus infection rates on early and late summer rice, both locally and within the insect migration range.

(2) Disease prevention via pest management. This strategy focuses on managing WBPH during the pre-jointing stages of early and late summer rice. Seedling planting locations and sowing times should be properly selected. Physical protection can be used to avoid or reduce WBPH invasions. In addition, seeds can be treated before sowing with coating agents or systemic pesticides, and chemical control of WBPH after seedling transplanting may be helpful.

(3) Resistant rice cultivar screening. At present, some WBPH-resistant cultivars are available for production use, and work on screening and breeding rice cultivars with SRBSDD-resistance is ongoing.

(4) Disease prevention in cropping operations. In practice, infected seedlings should be diagnosed and discarded during the early rice growth stages, diseased tillering plants in fields with infection rates of 3–20% should be replaced with healthy tillers split from uninfected plants, and severely diseased fields should be abandoned and replanted with other crops.

## TENDENCY OF OUTBREAK AND EPIDEMIC

The only currently-recognized WBPH-transmitted rice viral disease, SRBSDD has spread extensively since its discovery in 2001. After several years of viral source accumulation, a devastating disease outbreak occurred in northern Vietnam and southern China in 2009–2010, and although great efforts at disease management and vector control were made, the disease still damaged over 700,000 ha in 2011 and over 500,000 ha in 2012 in those two countries. During the past several years, the disease has spread to the rice growth region in southwestern China, and the virus has invaded the two major overwintering areas, the Gulf of Tonkin and Yunnan Province, China. It is predictable that SRBSDD will become a common major rice disease that occurs severely in limited locations and largely prevails in some years. The logic includes:

(1) The virus has an extensive area with a variety of conditions for overwintering. The virus is transmitted by WBPH in a persistent propagative manner and can be carried throughout the vector’s life, so it may overwinter in WBPH and in many plant species (including some weeds). Although the availability of these susceptible overwintering plants as sources of virus infection must still be experimentally examined, the viral overwintering range is probably even larger than the vector’s. Moreover, WBPH is more cold-resistant than BPH, overwinters in the vast warm areas south of 26° north latitude (i.e., southern Hainan Island and southwestern Yunnan Province), and propagates there year round. These conditions favor long-term survival of the virus, which maintains a high potential to trigger future outbreaks and epidemics of SRBSDD.

(2) The vector immigration period coincides strongly with the period of greatest rice susceptibility (the early growth stage), which may promote efficient secondary infection. As long-distance migrants, WBPH are very active and propagate rapidly; their populations can be many 10 to 100-folds within 1 month. More than 80% of the offspring feeding on infected plants will became viruliferous, and each individual can infect many plants. Obviously, even if only a small percentage of invasive WBPH are viruliferous, they might cause severe outbreaks of SRBSDD under favorable environmental conditions.

(3) The current rice cultivars and cropping systems are favorable for SRBSDD occurrence. To date, most of the main rice cultivars are susceptible to WBPH, and no SRBSDV-resistant cultivars have been found. Hybrid rice cultivars susceptible to SRBSDV and highly compatible with WBPH are widely grown in China. The seedling stage of summer rice in double-cropping regions temporally overlaps with the late growth stage of spring rice, and the two are spatially near, which is conducive to the transfer of the virus vector from spring to summer rice. Finally, the companion planting of single- and double-cropping rice in many areas with long sowing periods and the intercropping of maize also promote the translocation of SRBSDV and WBPH.

(4) Global warming might expand the overwintering region for SRBSDV and WBPH, increase the survival rate of overwintering vector, and result in more serious and widespread SRBSDV outbreaks.

(5) The disease is difficult to forecast and control. Only a dozen years have passed since SRBSDD was first recognized, and more studies are needed to better understand its epidemiology and develop monitoring technologies and control strategies. Exact forecasting of the disease is complicated by large uncertainties in the variable inter-year resources, immigration locations, invasion times and quantities, and infection rates of the migratory WBPH vector. Furthermore, because of the disease’s relatively long incubation period, the early symptoms are difficult to recognize.

In general, SRBSDD is likely to be more widespread and more serious in the near future, and therefore will become a great threat to rice production in Asia. Further studies on the epidemiology of the disease, on long-term monitoring systems and management technologies, on screening and breeding virus-resistant rice cultivars, and on control technology are urgently needed to combat this devastating disease.

## Conflict of Interest Statement

The authors declare that the research was conducted in the absence of any commercial or financial relationships that could be construed as a potential conflict of interest.
